# THE EFFECT OF COGNITIVE TRAINING AND PHYSICAL ACTIVITY PROGRAM IN COGNITIVE FUNCTION OF ELDERLY FACILITY RESIDENTS

**DOI:** 10.1093/geroni/igz038.3135

**Published:** 2019-11-08

**Authors:** Chien-Ning Tseng

**Affiliations:** Department of Nursing, Oriental Institute of Technology, Nil, Taiwan

## Abstract

This study evaluated the effectiveness of a combined cognitive training (CT) and physical activity (PA) intervention in improving cognitive function for institutionalized older residents with cognitive impairment. An experimental design with pre/post-test evaluations in a double-blind assessments at three points (baseline, T0; post-treatment, T1; 8-weeks-follow-up, T2), conducted an 8-week-CTPA intervention. Participants (N=134) were recruited from 12 institutions. Centers were randomly assigned into wait-list control, treatment I or treatment II groups. Treatment I group (low frequency) underwent combined 30-minutes sessions of individual-non-computer-based multi-domain CT (twice a week) with 30-minutes-group-chair-based PA (3 times a week). Treatment II group underwent the same protocol as Treatment I group, but with high frequency, 5 days per week for both CT and PA. The primary outcome, Cognitive Assessment Screening Instrument(CASI) total scores showed significant improvement in the treatment I and treatment II groups at T1-T0 and T2-T0, compared to the wait-list control group(10.55±9.60, 12.75±11.64, -8.01±6.61, p=0.000; 8.32±7.81, 11.75±10.19, -7.11±5.78, p=0.000), however there were no significant differences between two treatment groups. In CASI’s nine sub-domains, all the mean difference between groups were also significant at T1-T0 and T2-T0 (all p<0.05). The two treatment groups only significantly differ on CASI-ORIENT domain at T2-T0 (p=0.02). The findings revealed that a combined CT-and-PA intervention have positive immediate (T1-T0) and delayed (T2-T0) effects in cognitive function for older institutional residents with cognition-impairment. The two treatment groups did not show dose-response relationship. Even more, the low frequency intervention was more effective on several domains than high frequency intervention did.

